# Effectiveness of Myofascial Release Combined With Capacitive-Resistive Therapy in Patients With Chronic Nonspecific Low Back Pain: A Randomized Controlled Trial

**DOI:** 10.1155/prm/9309502

**Published:** 2025-07-11

**Authors:** Peng Zhao, Zhoupeng Lu, Hui Zou, Jialin Wang, Yuwei He, Meng Li, Jianfa Xu, Xinwen Cui

**Affiliations:** ^1^China Institute of Sport Science, Beijing, China; ^2^Beijing Sport University, Beijing, China; ^3^Chengdu Sport University, Chengdu, China

**Keywords:** chronic nonspecific low back pain, myofascial release, randomized controlled trial, TEACR therapy, thoracolumbar fascia

## Abstract

**Background:** Chronic nonspecific low back pain (CNLBP) is often associated with impaired mobility, functional limitations, and psychological distress. While myofascial release (MFR) and capacitive-resistive therapy (TECAR) have individually shown potential benefits, evidence regarding their combined application is limited.

**Methods:** This assessor-blinded, three-arm randomized controlled trial included 67 patients with CNLBP. Participants were assigned to MFR alone, resistive-mode TECAR (R-TECAR) alone, or MFR plus R-TECAR. Interventions were administered twice weekly for 4 weeks, with each session lasting 20 min. Primary outcomes included the Numeric Pain Rating Scale (NPRS) and the Roland–Morris Disability Questionnaire (RMDQ), assessed at the baseline, 4 weeks, and one-and-a-half-month follow-up. Secondary outcomes encompassed thoracolumbar fascia (TLF) thickness, pressure pain threshold (PPT), trunk mobility, quality of life, anxiety, and depression. Intention-to-treat analyses were performed.

**Results:** All interventions yielded significant improvements in pain and disability over time, although the combined MFR + R-TECAR therapy did not achieve statistically significant additional benefits compared with single therapies. Notably, a significant interaction effect emerged for PPT in the right quadratus lumborum muscle (*p*=0.01), with the MFR + R-TECAR group demonstrating greater improvement than R-TECAR alone. Other secondary outcomes, including TLF thickness and psychometric measures, improved over time but showed no significant between-group differences.

**Conclusions:** Combining MFR with R-TECAR for CNLBP did not produce superior outcomes compared with individual treatments though certain muscle-specific benefits were observed. Future research should focus on optimizing treatment parameters, extending intervention and follow-up periods, and exploring individualized approaches to maximize therapeutic efficacy.

**Trial Registration:** Chinese Registry of Clinical Trials: ChiCTR2400087961

## 1. Introduction

Chronic nonspecific low back pain (CNLBP) is characterized by pain between the 12th rib and the gluteal fold lasting more than 12 weeks without a specific underlying cause and affects individuals of all ages [1]. It is one of the leading causes of disability worldwide [1]. Patients with CNLBP often experience limited mobility, functional impairment, and psychological issues, imposing a significant burden on personal quality of life and healthcare systems [2, 3].

Although the exact pathogenesis of CNLBP remains unclear, recent studies suggest that the thoracolumbar fascia (TLF) may play a crucial role [4–6]. The TLF is a dense connective tissue rich in sensory nerve endings and nociceptors [4, 7]. Pathological changes in the TLF, such as reduced shear strain [5], increased thickness, and decreased sliding mobility [6], have been closely associated with chronic low back pain. In addition, the TLF's role in spinal stability and tension transmission further underscores its significance in pain mechanisms.

Myofascial release (MFR) is a fascia-oriented manual therapy (MT) commonly used in the management of low back pain [8]. Its proposed mechanisms include improving fascial mobility and reducing myofascial tension [9], as well as alleviating mechanical stress on pain-sensitive structures such as nerves and blood vessels by restoring the length and pliability of restricted connective tissue [10]. Recent clinical studies and systematic reviews suggest that MFR may provide short-term benefits in terms of pain reduction and functional improvement; however, its effects on spinal mobility and patient-reported quality of life remain inconclusive [8, 11, 12]. For example, while Arguisuelas et al. demonstrated significant improvements in pain and disability following four sessions of isolated MFR [12], Boff et al. reported conflicting results, showing minimal benefits [13]. These inconsistencies highlight the variability in study quality and methodology across the existing literature, indicating the need to explore optimized MFR protocols or potential synergistic effects through combination with other complementary therapies.

Capacitive-resistive therapy (TECAR) is a noninvasive electrothermal modality that operates in two distinct modes—capacitive and resistive—designed to target superficial and deep tissues, respectively [14]. The resistive mode has been shown to significantly enhance deep tissue perfusion [15] and contribute to pain relief in chronic musculoskeletal conditions [16]. Recent technological advancements allow therapists to apply TECAR using handheld electrodes, enabling precise and dynamic delivery of the intervention during manual contact [17]. Preliminary findings suggest that TECAR may improve pain, functional disability, and spinal range of motion in individuals with CNLBP [17]. However, high-quality randomized controlled trials investigating its standalone effectiveness in this population remain scarce, and the optimal therapeutic parameters and indications are yet to be fully established.

Currently, there is limited research on the efficacy of combining MFR with TECAR. Given their potentially complementary mechanisms—MFR facilitating fascial mobility and TECAR promoting deep tissue circulation—integrating these modalities may enhance clinical outcomes. This study aims to evaluate the relative effectiveness of MFR combined with resistive-mode TECAR (R-TECAR) in treating CNLBP. We hypothesize that the combined treatment will offer superior improvements in pain relief, functional recovery, and physiological structure of the TLF compared with using MFR or R-TECAR alone.

## 2. Materials and Methods

### 2.1. Study Design

This was a 4 week, assessor-blinded, three-arm randomized controlled trial with a one-and-a-half-month follow-up period. The study aimed to compare the effects of MFR combined with R-TECAR versus MFR alone and R-TECAR alone in patients with CNLBP. The study protocol was approved by the Ethics Committee of the National Institute of Sports Medicine. Written informed consent was obtained from all patients before inclusion.

Participants were recruited between August 7, 2024, and August 15, 2024, through WeChat advertisements. Due to recruitment time constraints, we ultimately enrolled 67 patients, leading us to modify the original four-arm design to a three-arm study. Follow-up assessments were completed on November 16, 2024.

### 2.2. Participants

  Inclusion criteria were (1) aged between 18 and 65 years; (2) localized pain between the 12th rib and the gluteal fold persisting for more than 12 weeks; (3) a baseline Numeric Pain Rating Scale (NPRS) score higher than three but less than eight; and (4) provided written informed consent.
  Exclusion criteria included (1) radicular pain extending into the lower limbs due to nerve root compression; (2) previous spinal surgery, trauma, or fractures; (3) implanted pacemaker; (4) pregnancy; (5) cancer; (6) systemic musculoskeletal diseases; (7) diagnosed neurodegenerative disorders (e.g., Parkinson's disease); (8) epilepsy; (9) history of psychiatric disorders; (10) skin infections or ulcers at the treatment site; and (11) receipt of MT applied to the lumbosacral region within 1 month before the intervention. To ensure the inclusion of patients with truly CNLBP, all participants were initially assessed by an experienced physical therapist. A thorough clinical assessment, including physical examination and review of medical history, was performed to exclude specific spinal pathologies. Only participants who met all inclusion criteria and none of the exclusion criteria were enrolled in the study.

### 2.3. Randomization and Blinding

Participants were randomly assigned to one of three groups: (1) MFR combined with R-TECAR therapy group, (2) MFR group, and (3) R-TECAR group. A stratified block randomization procedure with a 1:1:1 allocation ratio was implemented using R software (Version 4.1.1) and a reproducible script. Stratification factors included sex (male/female), age (18–45, > 45), and Body Mass Index (BMI) (< 25, ≥ 25) to ensure balanced allocation across key baseline characteristics. The randomization sequence was generated by an independent researcher who was not involved in recruitment, assessment, or intervention delivery. Group assignments were concealed in sequentially numbered, opaque, sealed envelopes, which were opened only after baseline assessments were completed. Outcome assessors were blinded to group assignments and did not participate in treatment delivery. Participants were instructed not to disclose their group allocation to assessors.

### 2.4. Participant Characteristics

Baseline demographic and clinical characteristics were collected through standardized questionnaires during the initial screening session. Age, sex, height, and weight were recorded and used to calculate BMI. Weekly work time was derived by multiplying the number of reported workdays per week by the average number of working hours per day. Educational attainment was classified into two categories: high school/middle school and undergraduate/college or above. Exercise status was determined based on self-reported physical activity over the past month. Participants who reported engaging in any form of exercise at least twice per week, for a minimum of 30 min per session, were classified as physically active. Sleep duration was self-reported and categorized as < 6 h, 6–8 h, or > 8 h per night. Daily sitting time (in hours) was also recorded. Other pain captured the presence of musculoskeletal pain in areas other than the low back (e.g., neck, shoulder, or knees) within the last 3 months. Pain position was determined by asking participants and categorized as left sided, right sided, bilateral, or central. Pain duration (referred to as “pain year”) was defined as the total number of years the participant had experienced low back pain symptoms prior to enrollment.

### 2.5. Outcome Measures

Experienced therapists conducted measurements at the baseline (before the intervention), after 4 weeks (end of the intervention), and at the one-and-a-half-month follow-up. The primary outcome measures were pain intensity and the Roland–Morris Disability Questionnaire (RMDQ). Secondary outcomes included TLF thickness, pressure pain threshold (PPT), trunk mobility, anxiety and depression levels, overall improvement, and quality of life. Pain intensity, disability, quality of life, anxiety, and depression were assessed at baseline, 4 weeks, and follow-up, while TLF thickness, PPT, and trunk mobility were measured at the baseline and after 4 weeks.

### 2.6. Primary Outcome Measures

#### 2.6.1. Pain Intensity

Pain intensity was assessed using the NPRS, an 11-point scale ranging from 0 (“no pain”) to 10 (“worst imaginable pain”) [18]. Participants reported their worst pain, least pain, and current pain over the past 24 h; the average of these three values was used as the pain intensity score. The NPRS has demonstrated high reliability (ICC: 0.80–0.95) and validity in populations with low back pain [18, 19].

#### 2.6.2. RMDQ

The RMDQ is a 24-item questionnaire designed to assess disability related to daily activities in individuals with chronic low back pain [20]. Scores range from 0 to 24, with higher scores indicating greater disability [20]. The RMDQ is highly sensitive for measuring disability in low back pain patients, offering advantages such as simplicity and ease of understanding [21]. The simplified Chinese version of the RMDQ has shown excellent reliability and validity in Chinese-speaking populations [22].

#### 2.6.3. Secondary Outcome Measures

Secondary outcomes encompass TLF thickness, PPT, trunk mobility, health-related quality of life, and psychological status. TLF thickness was measured bilaterally at 2 cm lateral to the L2–L3 interspinous level using musculoskeletal ultrasound (MIP08 Portable Color Doppler Ultrasound System, KiviSonic series, China), ensuring consistent settings and following protocols with high inter-rater reliability [6, 23]. PPT was assessed at the bilateral quadratus lumborum and paraspinal muscles at the L4-L5 interspinous space using a digital algometer (Wagner Instruments, Greenwich, CT, USA), with measurements averaged over three trials to enhance accuracy [24, 25]. Trunk mobility was evaluated using the fingertip-to-floor (FTF) test, a validated measure where participants reached toward the floor to assess spinal flexibility [26, 27]. Health-related quality of life was measured using the EuroQol Five Dimensions Questionnaire-5L (EQ-5D-5L), covering mobility, self-care, usual activities, pain/discomfort, and anxiety/depression, and validated in low back pain populations [28–30]. Psychological status was evaluated using the Patient Health Questionnaire-9 (PHQ-9) and the Generalized Anxiety Disorder-7 (GAD-7), both reliable and valid tools for assessing depression and anxiety in Chinese patients with chronic low back pain [31, 32].

### 2.7. Experimental Protocols

MFR techniques were applied according to the methods described in “Fascial Release for Structural Balance” by Myers and Earls [33]. Treatment targeted the bilateral iliac fascia, psoas major, lateral raphe, erector spinae, multifidus, quadratus lumborum fascia, and TLF. For each target region, low-load sustained pressure was applied in the direction of fascial restriction for approximately 90–120 s, with 2-3 repetitions per area. To enhance the specificity and effectiveness of the release, patients were instructed to perform active movements—such as anterior–posterior pelvic tilts—when treating the iliopsoas region. Intervention sessions were conducted twice a week, each lasting 20 min, with a 72-h interval between sessions, over a total period of four weeks.

For the R-TECAR group, therapy was delivered using the WinBack TECAR device (WINBACK 3SE, Villeneuve Loubet, France) in resistive mode with a frequency of 500 kHz. A flexible, self-adhesive return electrode (10 cm × 18 cm) was placed on the abdomen as a reference electrode. The power output was gradually increased from approximately 30%–60% of the device's maximum output (100 W), with adjustments based on patient-reported thermal feedback to ensure a consistent perception of mild to moderate warmth. Each quadrant of the lumbar region was treated for approximately 5 min, totaling 20 min per session.

Participants in the combined MFR + R-TECAR group received the same MFR protocol described above, integrated with R-TECAR therapy. The parameters for R-TECAR in this group, including frequency, intensity, and electrode placement, were identical to those used in the R-TECAR group. This ensured consistency in the application of R-TECAR across groups, allowing for a precise evaluation of the additive effects of combining MFR and R-TECAR.

### 2.8. Sample Size Determination

An a priori power analysis was performed using G∗Power software (Version 3.1.9.7) [34] to determine the minimum sample size required for this study. The calculation was based on a two-tailed significance level (*α* = 0.05), a statistical power of 80% (1–β = 0.80), and an anticipated effect size of *f* = 0.38 (Cohen's *f*), which was derived from the between-group differences in pain intensity reported by the previous study [17]. Specifically, in that study, the NPRS score 2 weeks after treatment was 3.2 (SD = 1.24) in the group receiving combined TECAR and MT, compared with 4.1 (SD = 1.11) in the MT group alone. These values yielded a Cohen's d of approximately 0.76, which was subsequently converted to Cohen's f for the purpose of analysis of variance (ANOVA)-based estimation.

Based on these parameters, the minimum sample size required to detect a statistically significant between-group difference was calculated to be 44 participants. To account for a potential attrition rate of 25% during the follow-up period, the target sample size was adjusted to 59 participants.

### 2.9. Statistical Analysis

All statistical analyses were performed using R software (Version 4.4.1) based on the intention-to-treat (ITT) principle. Missing data were handled using multiple imputations via the “mice” package in R to minimize potential bias. Five imputed datasets were generated using predictive mean matching (PMM) across 50 iterations, under the assumption that data were missing at random (MAR). The imputation model included age, sex, BMI, and baseline values of the outcome variables as predictors. Continuous baseline variables were presented as mean with 95% confidence intervals (CIs), and normality was assessed using the Shapiro–Wilk test. For normally distributed data, between-group comparisons were conducted using one-way ANOVA, while the Kruskal–Wallis H test was used for non-normally distributed data. Categorical variables were summarized as frequencies and percentages, and Fisher's exact test was employed for group comparisons.

Outcome measures were analyzed using two-way repeated measures ANOVA via the “ez” package in R to evaluate the interaction effects of “group” and “time.” Post hoc analyses used Bonferroni correction to examine simple main effects when significant interactions were observed. All statistical tests were two sided, with a significance level set at *p* < 0.05.

## 3. Results

**Figure 1:**
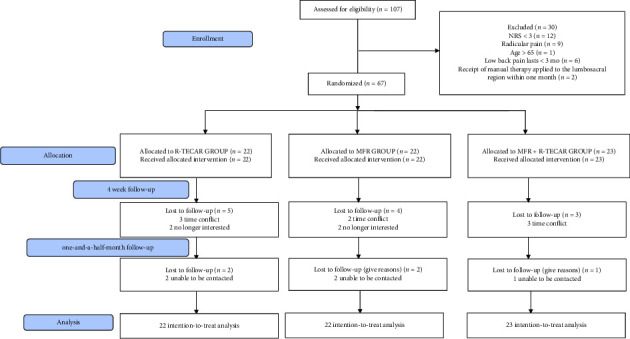
CONSORT flow diagram of the study.

In August 2024, 107 potential participants were screened for eligibility. Sixty-seven individuals met all inclusion criteria and were randomly assigned to one of three groups: the MFR group (*n* = 22), the R-TECAR group (*n* = 22), and the combined MFR + R-TECAR group (*n* = 23) (Figure 1). Of these participants, 50 (74.6%) completed the one-and-a-half-month follow-up. The overall mean age of the participants was 40.2 ± 10 years, and 39 (58.2%) were female. Detailed demographic and baseline characteristics for each group are presented in Table 1.

### 3.1. Primary Outcome Measures

Table 2 summarizes the results of the primary outcome measures. There was a significant improvement over time in both the NPRS and RMDQ scores across all groups (time effect, *p* < 0.01), indicating that all three interventions—MFR, R-TECAR, and combined MFR + R-TECAR—were effective in reducing pain and improving function. Between-group differences in NPRS scores did not reach statistical significance (*p*=0.13). However, the between-group effect for RMDQ scores approached significance (*p*=0.07), suggesting a trend toward greater functional improvement in MFR + R-TECAR. No significant interaction effects were observed between group and time for either NPRS or RMDQ scores (interaction effect, *p* > 0.05).

### 3.2. Secondary Outcome Measures

The results of the secondary outcome measures are presented in Table 3. Significant improvements over time were observed in most secondary outcomes, including EQ-5D-5L, PHQ-9, TLF thickness, FTF test scores, and PPT of the quadratus lumborum muscle (time effect, *p* < 0.01). This indicates that all interventions contributed to enhancements in physical function and psychological wellbeing.

Between-group differences were not statistically significant for most secondary outcomes. However, a significant between-group difference was found in depression scores measured by the PHQ-9 (*p* < 0.01), with the combined MFR + R-TECAR group showing greater improvement than the other groups.

Notably, a significant interaction effect was observed for the PPT of the right quadratus lumborum muscle (interaction effect, *p* < 0.05), suggesting that the combined MFR + R-TECAR intervention may have specific advantages in increasing pain thresholds in this muscle. Post hoc Bonferroni correction revealed that the right quadratus lumborum PPT improvement was significantly greater in the combined MFR + R-TECAR group than in the R-TECAR group alone (*p*=0.01). In contrast, the PPTs of the bilateral paraspinal muscles at the L4–L5 interspinous space did not show significant changes over time (left side: *p*=0.43 and right side: *p*=0.21) between groups (left side: *p*=0.75 and right side: *p*=0.21), or in interaction effects (left side: *p*=0.34 and right side: *p*=0.46).

## 4. Discussion

Our ITT analysis over a 1-month intervention period and a one-and-a-half-month follow-up demonstrated that combining MFR with R-TECAR did not result in statistically significant additional improvements in pain reduction and functional enhancement for patients with CNLBP compared with MFR or R-TECAR alone. Notably, we observed a significant interaction effect in the PPT of the right quadratus lumborum muscle among secondary outcomes. Post hoc analysis revealed that the MFR + R-TECAR group exhibited a significantly greater improvement in PPT compared with the R-TECAR group alone. The other secondary outcome measures did not show statistically significant differences among the groups.

Regarding the primary outcomes, between-group differences in the NPRS scores at both postintervention and follow-up time points were modest. Specifically, the MFR + R-TECAR group demonstrated differences of 0.70 and 0.43 points (vs. MFR and R-TECAR, respectively) after the 4-week intervention and 0.44 and 1.20 points at the follow-up. All of these values were below the established minimal clinically important difference (MCID) of two points [35], and the corresponding 95% CIs overlapped with comparator groups, indicating neither statistical nor clinical significance. Similarly, between-group differences in RMDQ scores were 1.85 and 2.21 points (postintervention) and 1.66 and 3.11 points (follow-up) for comparisons between MFR + R-TECAR and MFR or R-TECAR, respectively. These values fell below the MCID of five points [36] for functional disability and were likewise associated with overlapping 95% CI, suggesting a lack of robust evidence for the superiority of the combined approach.

Our findings contrast with those of Kasimis et al. [17], who demonstrated that combining TECAR with MT soft tissue techniques led to better outcomes than MT alone. A possible reason for this discrepancy is that they utilized traditional capacitive electrodes for lumbar treatment during the combined TECAR whereas our study employed R-TECAR. In addition, their intervention duration may have been longer whereas our study provided only a 20-min intervention per session. Another study investigating TECAR combined with conventional treatment for upper trapezius myofascial pain syndrome found no additional benefit in improving shoulder disability [16], which aligns with our findings regarding disability measures. However, they reported better outcomes in pain relief and other aspects. Since they did not use modern specialized electrodes, direct comparisons with our results are challenging.

The significant improvement in the PPT of the right quadratus lumborum muscle suggests that the combined MFR and R-TECAR intervention may have specific advantages in increasing pain thresholds in this muscle. This effect was not observed in the left quadratus lumborum muscle or the bilateral paraspinal muscles at the L4-L5 interspinous space. This asymmetry may be due to subtle differences in neural innervation, blood supply, or muscle fiber composition between the left and right quadratus lumborum muscles [37, 38]. In addition, during treatment, the therapist may inadvertently pay more attention to the right side, resulting in better results on the right side than on the left side.

MFR has been shown to improve the pathological conditions of the TLF [9]. In our study, ultrasound measurements revealed that both therapies significantly reduced the thickness of the bilateral TLF after treatment, providing new insights for future treatments of fascia-related disorders. Moreover, both MFR and R-TECAR can enhance microcirculation in deep tissues [15, 39], which may play a crucial role in ameliorating the pathological state of the TLF. However, combining MFR with R-TECAR did not yield superior results compared with the individual therapies in most outcome measures. This may be because both interventions act through similar physiological mechanisms—improving deep tissue blood circulation [15, 39]. When combined, their effects might overlap, failing to produce the expected synergistic benefits.

The lack of significant additional benefits from the combined therapy may also be attributed to the dosage and duration of the interventions. Our treatment sessions were limited to 20 min, which may not have been sufficient to elicit maximal therapeutic effects.

### 4.1. Limitations

This study has several limitations that should be acknowledged. First, the sample size was relatively modest (*n* = 67), and only 74.6% of the participants completed all outcome assessments during the one-and-a-half-month follow-up period. This attrition and limited sample size may have reduced the statistical power to detect subtle but clinically meaningful differences. Second, the intervention period was relatively short, with each session lasting only 20 min across 4 weeks, and the follow-up period was limited to 6 weeks postintervention. These timeframes may not have been sufficient to capture the full therapeutic potential or long-term sustainability of the interventions. Third, we evaluated only a limited number of outcome measures and targeted specific muscles, which may not comprehensively represent the broader neuromuscular and functional impacts of the treatments on the lumbar region. Finally, participants were recruited from the general community rather than clinical care settings and were not actively seeking medical treatment at the time of enrollment. This population may differ from typical treatment-seeking patients in terms of symptom severity, healthcare motivation, and adherence behavior, potentially limiting the generalizability of our findings to broader clinical populations. Future studies with larger sample sizes, longer follow-up periods, and recruitment from clinical populations are warranted to confirm and extend our findings.

## 5. Conclusions

This study evaluated the efficacy of combining MFR with R-TECAR in patients with CNLBP. The results indicated that the combined therapy was not significantly superior to MFR or R-TECAR alone in terms of pain relief and functional improvement. Future research should aim to optimize the parameters of the combined therapy and explore individualized treatment strategies to enhance therapeutic efficacy.

## Figures and Tables

**Table 1:** Baseline characteristics.

Level	Overall	MFR	MFR + R-TECAR	R-TECAR	*p*
*N*	67	22	23	22	

Age (mean (SD)), years	40.2 (10.0)	40.9 (11.2)	40.8 (8.8)	38.8 (10.2)	0.738

*Gender (%)*					
Male	28 (41.8)	10 (45.5)	9 (39.1)	9 (40.9)	0.907
Female	39 (58.2)	12 (54.5)	14 (60.9)	13 (59.1)	

BMI (mean (SD))	22.8 (3.0)	22.1 (2.9)	23.4 (2.9)	23.0 (3.1)	0.341

Every week work time (mean (SD)), h	29.4 (17.7)	30.2 (18.8)	31.3 (17.0)	26.7 (17.6)	0.668

*Education (%)*					
High school/middle school	2 (3.0)	0 (0.0)	0 (0.0)	2 (9.1)	0.121
Undergraduate/college or above	65 (97.0)	22 (100.0)	23 (100.0)	20 (90.9)	

*Other pain (%)*					
No	23 (34.3)	8 (36.4)	8 (34.8)	7 (31.8)	0.949
Yes	44 (65.7)	14 (63.6)	15 (65.2)	15 (68.2)	

*Exercise (%)*					
No	33 (49.3)	12 (54.5)	13 (56.5)	8 (36.4)	0.334
Yes	34 (50.7)	10 (45.5)	10 (43.5)	14 (63.6)	

*Sleep (%)*					
More than 8 h	6 (9.0)	1 (4.5)	3 (13.0)	2 (9.1)	0.243
6–8 h	54 (80.6)	21 (95.5)	16 (69.6)	17 (77.3)	
Less than 6 h	7 (10.4)	0 (0.0)	4 (17.4)	3 (13.6)	

Sitting time (mean (SD)), h	6.3 (2.0)	6.4 (2.0)	6.0 (2.3)	6.6 (1.8)	0.618

*Pain position (%)*					
Left	7 (10.4)	2 (9.1)	4 (17.4)	1 (4.5)	0.17
Right	10 (14.9)	6 (27.3)	3 (13.0)	1 (4.5)	
Both side	49 (73.1)	14 (63.6)	15 (65.2)	20 (90.9)	
Middle	1 (1.5)	0 (0.0)	1 (4.3)	0 (0.0)	

Pain year (mean (SD)), years	3.8 (5.1)	3.3 (5.9)	3.9 (4.9)	4.2 (4.6)	0.85

*Note:* R-TECAR: resistive-mode TECAR therapy; MFR: myofascial release.

**Table 2:** The result of primary outcome.

Timepoint	R-TECAR (mean ± SD)	MFR (mean ± SD)	MFR + R-TECAR (mean ± SD)	Mean difference (95% CI): (MFR + R-TECAR)—R-TECAR	Mean difference (95% CI): MFR—(MFR + R-TECAR)	Mean difference (95% CI): MFR—TECAR_R	*p*-value (group)	*p*-value (time)	*p*-value (interaction)
*NRS score (0–10)*									
Baseline	4.62 ± 1.41	4.08 ± 1.17	4.09 ± 1.11	−0.53 (−1.66, 0.59)	−0.01 (−1.14, 1.11)	−0.55 (–1.69, 0.59)	0.13	< 0.01	0.27
Week 4	3.00 ± 1.77	3.27 ± 1.32	2.57 ± 1.83	−0.43 (−1.56, 0.69)	0.71 (−0.42, 1.83)	0.27 (−0.86, 1.41)			
Follow-up	3.61 ± 1.77	2.85 ± 1.70	2.41 ± 2.01	−1.20 (−2.32, −0.08)	−0.44 (−0.68, 1.57)	−0.76 (−1.89, 0.38)			

*RMDQ score (0–24)*									
Baseline	6.23 ± 3.01	6.91 ± 3.91	5.87 ± 3.51	−0.36 (−2.72, 2.02)	−1.04 (−1.32, 3.40)	0.68 (−1.70, 3.07)	0.07	< 0.01	0.06
Week 4	5.86 ± 2.64	5.50 ± 3.73	3.65 ± 2.59	−2.21 (−4.57, 0.147)	1.85 (-0.51, 4.21)	−0.36 (−2.75, 2.02)			
Follow-up	6.50 ± 3.25	5.05 ± 4.36	3.39 ± 2.73	−3.11 (−5.47, −0.75)	1.66 (0.70, 4.01)	−1.46 (−3.84, 0.93)			

*Note:* R-TECAR: resistive-mode TECAR; MFR: myofascial release; NRS: Numeric Pain Rating Scale.

Abbreviation: RMDQ, Roland–Morris Disability Questionnaire.

**Table 3:** The result of secondary outcome.

Timepoint	R-TECAR (mean (SD))	MFR (Mean (SD))	MFR + R-TECAR (mean (SD))	Mean difference (95% CI): (MFR + R-TECAR)—R-TECAR	Mean difference (95% CI): MFR—(MFR + R-TECAR)	Mean difference (95% CI): MFR—TECAR_R	*p* value (group)	*p* value (time)	*p* value (interaction)
*Left TLF thickness (cm)*									
Baseline	0.33 (0.16)	0.33 (0.21)	0.36 (0.19)	0.04 (−0.07, 0.14)	−0.04 (−0.14, 0.07)	0.00 (−0.10, 0.11)	0.87	< 0.01	0.58
Week 4	0.26 (0.10)	0.25 (0.07)	0.25 (0.10)	−0.01 (−0.11, 0.98)	0.00 (−0.10, 0.11)	−0.01 (−0.11, 0.10)			

*Right TLF thickness (cm)*									
Baseline	0.38 (0.18)	0.37 (0.25)	0.36 (0.16)	−0.03 (−0.14, 0.09)	0.01 (−0.10, 0.13)	−0.14 (−0.13, 0.10)	0.83	< 0.01	0.69
Week 4	0.27 (0.11)	0.30 (0.09)	0.26 (0.12)	−0.01 (−0.12, 0.11)	0.04 (−0.08, 0.15)	0.03 (−0.08, 0.15)			

*GAD-7 (0–7)*									
Baseline	3.86 (2.75)	3.00 (2.49)	2.96 (2.27)	−0.91 (−2.58, 0.77)	0.044 (−1.63, 1.72)	−0.86 (−2.55, 0.83)	0.06	0.05	0.65
Week 4	3.32 (2.77)	2.50 (2.30)	2.17 (2.19)	−1.14 (−2.82, 0.63)	0.33 (−1.35, 2.00)	−0.82 (−2.51, 0.87)			
Follow-up	3.82 (2.89)	2.27 (1.86)	1.91 (1.59)	−1.01 (−3.58, −0.23)	0.36 (−1.31, 2.03)	−1.55 (−3.24, 0.15)			

*PHQ-9 (0–9)*									
Baseline	6.45 (3.36)	3.59 (2.46)	4.43 (2.81)	−2.02 (−4.11, 0.07)	−0.84 (−2.94, 1.25)	−2.86 (−4.98, −0.75)	< 0.01	< 0.01	0.50
Week 4	5.45 (3.16)	3.45 (2.58)	3.65 (1.90)	−1.80 (−3.89, 0.29)	−0.20 (−2.29, 1.89)	−2.00 (−4.11, 0.11)			
Follow-up	5.36 (3.57)	2.91 (3.25)	2.61 (3.29)	−2.76 (−4.85, −0.66)	0.30 (−1.79, 2.39)	−2.46 (−4.57, −0.34)			

*EQ-5D-5L (0–100)*									
Baseline	0.82 (0.12)	0.83 (0.16)	0.82 (0.12)	0.00 (−0.07, 0.08)	0.01 (−0.07, 0.08)	0.01 (−0.07, 0.09)	0.29	< 0.01	0.35
Week 4	0.87 (0.08)	0.89 (0.09)	0.89 (0.07)	0.02 (−0.05, 0.10)	0.00 (−0.08, 0.07)	0.03 (−0.05, 0.10)			
Follow-up	0.83 (0.11)	0.89 (0.11)	0.91 (0.10)	0.08 (0.00, 0.16)	−0.03 (−0.10, 0.05)	0.05 (−0.02, 0.13)			

*FTF (cm)*									
Baseline	9.65 (8.44)	11.8 (10.8)	11.9 (10.4)	2.29 (−3.97, 8.54)	−0.12 (−6.37, 6.14)	2.17 (−4.15, 8.50)	0.81	0.02	0.23
Week 4	9.31 (6.63)	10.3 (8.31)	8.22 (7.75)	−1.10 (−7.35, 5.61)	2.07 (−4.19, 8.33)	0.98 (−5.35, 7.30)			

*L4-L5 paraspinal intervertebral space PPT,kg*/*cm*^2^*left*									
Baseline	3.80 (1.70)	3.50 (1.59)	3.43 (1.98)	−0.37 (−1.49, 0.74)	0.07 (−1.04, 1.18)	−0.30 (−1.43, 0.82)	0.75	0.43	0.34
Week 4	3.62 (1.45)	4.05 (1.32)	3.54 (1.31)	−0.08 (−1.12, 1.04)	0.50 (−0.61, 1.62)	0.42 (−0.70, 1.55)			

*L4-L5 paraspinal intervertebral space PPT,kg*/*cm*^2^*right*									
Baseline	3.65 (1.70)	3.47 (1.62)	3.35 (1.91)	−0.30 (−1.43, 0.84)	0.13 (−1.01, 1.26)	−0.17 (−1.32, 0.98)	0.72	0.21	0.46
Week 4	3.66 (1.50)	4.05 (1.50)	3.50 (1.35)	−0.16 (−1.30, 0.98)	0.55 (−0.59, 1.69)	0.39 (−0.76, 1.54)			

*Quadratus lumborum muscle PPT,kg*/*cm*^2^*left*									
Baseline	1.71 (0.71)	1.70 (0.79)	1.70 (0.92)	−0.01 (−0.54, 0.51)	−0.00 (−0.54, 0.53)	−0.02 (−0.55, 0.52)	0.74	< 0.01	0.47
Week 4	18.3 (0.76)	21.3 (0.74)	20.1 (0.54)	0.30 (−0.23, 0.83)	−0.12 (−0.65, 0.41)	0.18 (−0.36, 0.72)			

*Quadratus lumborum muscle PPT,kg*/*cm*^2^*right*									
Baseline	1.79 (0.89)	1.52 (0.67)	1.54 (0.72)	−0.25 (−0.76, 0.26)	−0.02 (−0.53, 0.49)	−0.27 (−0.79.0.24)	0.88	< 0.01	0.01
Week 4	1.67 (0.72)	1.87 (0.60)	2.04 (0.68)	0.37 (−0.14, 0.88)	−0.17 (−0.69, 0.34)	0.20 (−0.31, 0.71)			

*p* value (between groups): *p* : 0.01^a^									

*Note:* Between-group comparison: ^a^Group 1 versus group 3. R-TECAR: resistive-mode TECAR; MFR: myofascial release; TLF: thoracolumbar fascia; EQ-5D-5L: EuroQol Five Dimensions Questionnaire-5L; FTF: fingertip-to-floor test.

Abbreviations: GAD-7, Generalized Anxiety Disorder-7; PHQ-9, Patient Health Questionnaire-9; PPT, Pressure Pain Threshold.

## Data Availability

The data that support the findings of this study are available on request from the corresponding author. The data are not publicly available due to privacy or ethical restrictions.

## Nomenclature

CNLBP: Chronic nonspecific low back pain
MFR: Myofascial release
TECAR: Capacitive-resistive therapy
R-TECAR: Resistive-mode TECAR
BMI: Body Mass Index
NPRS: Numeric Pain Rating Scale
RMDQ: Roland–Morris Disability Questionnaire
TLF: Thoracolumbar fascia
PPT: Pressure pain threshold
FTF: Fingertip-to-floor
EQ-5D-5L: EuroQol Five Dimensions Questionnaire-5L
PHQ-9: Patient Health Questionnaire-9
GAD-7: Generalized Anxiety Disorder-7
BMI: Body Mass Index
SD: Standard deviation
MT: Manual therapy
ANOVA: Analysis of variance
ITT: Intention-to-treat
CI: Confidence interval
PMM: Predictive mean matching
